# Neuroglobin in Retinal Neurodegeneration: A Potential Target in Therapeutic Approaches

**DOI:** 10.3390/cells10113200

**Published:** 2021-11-17

**Authors:** Virginia Solar Fernandez, Maria Marino, Marco Fiocchetti

**Affiliations:** 1 Department of Science, University Roma Tre, Viale G. Marconi, 00146 Rome, Italy; virginia.solarfernandez@uniroma3.it (V.S.F.); maria.marino@uniroma3.it (M.M.); 2 Neuroendocrinology, Metabolism, and Neuropharmacology Unit, IRCCS Santa Lucia Foundation, 00143 Rome, Italy

**Keywords:** neuroglobin, retinal neurodegeneration, stress response

## Abstract

Retinal neurodegeneration affects an increasing number of people worldwide causing vision impairments and blindness, reducing quality of life, and generating a great economic challenge. Due to the complexity of the tissue, and the diversity of retinal neurodegenerative diseases in terms of etiology and clinical presentation, so far, there are no cures and only a few early pathological markers have been identified. Increasing efforts have been made to identify and potentiate endogenous protective mechanisms or to abolish detrimental stress responses to preserve retinal structure and function. The discovering of the intracellular monomeric globin neuroglobin (NGB), found at high concentration in the retina, has opened new possibilities for the treatment of retinal disease. Indeed, the NGB capability to reversibly bind oxygen and its neuroprotective function against several types of insults including oxidative stress, ischemia, and neurodegenerative conditions have raised the interest in the possible role of the globin as oxygen supplier in the retina and as a target for retinal neurodegeneration. Here, we provide the undercurrent knowledge on NGB distribution in retinal layers and the evidence about the connection between NGB level modulation and the functional outcome in terms of retinal neuroprotection to provide a novel therapeutic/preventive target for visual pathway degenerative disease.

## 1. Introduction

Globally, in 2015, it was estimated that blindness and moderate/severe vision impairments affect more than 36 and 216 million of people, respectively, and the number has raised along with the increase in population and age [1,2]. Retinal neurodegenerative diseases, including diabetic retinopathy, DR, retinitis pigmentosa, RP, age-related macular degeneration, AMD, or glaucoma, are the most frequent causes of irreversible vision impairment and blindness worldwide [3,4]. Despite the high economic and social impact of vision impairment and blindness, there are no cures for retinal neurodegenerative pathologies [4,5]. So far, the current approaches for the developing of new possible therapeutics rely on the definition of common pathways of degeneration to establish preventive intervention able to manipulate such intracellular pathways and counteract the disease mechanisms. Other strategies attempt to prevent the excessive activation of neuronal stress response including apoptotic activation and inflammatory events, or to replace retinal cells via stem cell transplantation or artificial vision [5]. In parallel, “time window” for treatment represents a critical aspect for retinal degeneration considering the evidence that a preventive strategy able to preserve functional cell and neuronal circuitries still in place is a more affordable and successful way for treatment in spite of any kind of other intervention at advanced degenerative stages [5]. It is becoming clear that a better knowledge of molecular pathways behind the protective or detrimental retinal cell stress response and the identification of early biological markers is required to improve the therapy devoted to tailored personal needs [5,6]. Over the last 20 years, the identification of neuroglobin (NGB) as an intracellular monomeric globin preferentially expressed in neurons which functions as endogenous cell protectant, has defined a new possible target for therapeutic intervention in neuronal pathologies spanning from ischemic injuries to neurodegeneration [7,8]. Since its discovering in 2000 [9], the identification that hypothalamus and retinal cells express the NGB at the highest level than other brain areas, have led to consider this globin as a possible oxygen carrier protein in the retina, raising further interest about the globin role in the retinal physiology and on its neuroprotective potential in the visual system. Here, we will discuss the up-to-date knowledge on NGB distribution in retinal layers and the evidence about the connection between NGB modulation by endogenous or exogenous events and the functional outcome in terms of retinal neuroprotection to provide a novel therapeutic/preventive target for visual pathway degenerative disease.

## 2. Retina and Retinal Degeneration

The retina is a high-differentiated nerve light-sensitive tissue that has been estimated to be responsible for the 80% of all sensory information in humans [5,10]. Functionally, the retina is a complex multilayer of interconnected neurons at the innermost levels of the eye globe, and it represents the beginning of the visual pathway receiving and translating the light information into neuronal signals and sending them via the optical nerve to the cortex of the brain where the visual processing takes place [5,11,12]. The retina is constituted by different types of cellular components including the retinal pigment epithelium (RPE) cells, neuronal and glial cells. Glial cells are divided into Müller cells, microglia, and astrocytes. Neuronal cells are organized into 5 major classes distributed into different layers with specific functions: photoreceptors (rods and cones) which represent the photosensitive cells of the retina, bipolar cells, horizontal cells, and amacrine cells forming the neuroretinal circuitries that transmit the signal from photoreceptors to ganglion cells responsible to transmit the visual information to the brain [5,10]. Histologically, the retina is divided into 10 layers constituting the outer retina and the inner retina. The outer retina comprises the RPE, the photoreceptor layer (PL) consisting of the outer (OSs) and inner segments (ISs), the layer of nuclei of photoreceptor cells (outer nuclear layer—ONL), and the middle of the outer plexiform layer (OPL) where the synapsis between photoreceptors and the bipolar and horizontal cells take form [12,13]. The inner retina includes the inner part of OPL, the inner nuclear layer (INL) constituted by the nuclei of bipolar, amacrine, horizontal, and Müller cells, the inner plexiform layer (IPL) where bipolar, amacrine, and ganglion cells connect each other, the ganglion cell layer (GCL) containing the nuclei of retinal ganglion cells (RGCs), the nerve fiber layer (NFL), predominantly formed by the axons of ganglion cells, retinal vessel, and glial cells, and the inner limiting membrane (ILM) [12,13].

Retina metabolism is the highest of the total central nervous system and the lack of oxygen under hypoxic conditions is thought to have a direct detrimental effect on visual function and to be pivotal for retinal disease [14,15,16]. The retina of mouse, man, and other mammals is nourished by two different vascular systems, a vascular plexus from the central retina artery (from the inner side of the retina) and choriocapillaris (from the outer side of the retina) separated from the RPE by the Bruch’s membrane, an elastic membrane composed by five layers [10,12,13,16]. In this particular context, the retina developed complex mechanisms to cope with the lack of vessels in the most energy-consuming area [17].

The complex structural and functional nature of the retina makes such tissue extremely vulnerable to aberration due to any kind of injury [3,5]. In fact, the retina has to respond to a series of environmental insults to whom is commonly exposed, including light-induced stress and oxidative stress and, because of the high metabolic activity of the tissue, it is particularly susceptible to variation of oxygen and nutrient availability [5].

Retinal neurodegenerative diseases are different pathologies in terms of etiology and clinical presentation [5]. Affecting 1 in 4000 people worldwide and being the principal cause of complete vision loss, RP constitutes a family of genetic retinal diseases that can add up to 250 gene mutations [18]. During the early stages, the patients suffer a reduction in night vision and peripheral vision due to the increased apoptotic death rate of rod photoreceptors, as the disease progresses eyesight becomes poorer and reduced to the central vision field, leading, eventually, to blindness [19]. A more complex disease is AMD, which commonly affects central vision. Initiated by a lipid and protein accumulation in the Bruch’s membrane, these form extracellular deposits known as drusen in the space between the RPE and the membrane, that gradually induce geographic atrophy characterized by choroid layer neovascularization and angiogenesis (only present on the wet form of the disease), loss of RPE, and, ultimately, by photoreceptors death [20]. On the other side, diseases affecting ganglion cells, such as glaucoma, may leave photoreceptors intact and completely functional, but the transmission of the visual information for processing and interpretation is impaired [21]. Though the exact cause of glaucoma is yet to be elucidated, the increase in the intraocular pressure has been suggested as one of the primary risk factors [22] resulting in an ischemic event that induces apoptosis of the RGC axons at the beginning of the optic nerve. However, other possible factors that can favor glaucoma include metabolic stress, neuroinflammation [23], and aging [24]. Diabetic retinopathy represents a group of ocular diseases, which could develop in diabetic people characterized by the change in microvascular structure and the presence of more fragile capillaries accompanied or not by neo-angiogenesis [5,6].

Despite differences in terms of etiology across the retinal neurodegenerative disease, among them, the molecular and cellular mechanisms of degeneration show a large pattern of similarity [3,4,5]. Independently from the etiology of the disease, the imbalance of the equilibrium between production and detoxification of reactive oxygen species (ROS) is considered the main contributor to the degeneration of the retina [3,5]. So that, genetic or environmental conditions that increase ROS production might lead to retinal neurodegeneration and the activation of antioxidant machinery is a common response mechanism triggered by stress at the level of both neuronal and glial cells of the retinal tissue. Aside, the deregulated activation of programmed cell death and the inflammatory response account for the morphological and functional impairments of retinal tissue [5].

## 3. Neuroglobin in the Retina

The brain was believed to lack any system able to promote the storage and/or the diffusion of oxygen similar to what occurs in the muscle system with myoglobin (MB). However, the discovery of NGB as a monomeric intracellular globin, that has been so named for its preferential expression in the central and peripheral nervous system, has raised the possibility of a myoglobin-like function of NGB [9,25]. NGB shows the typical globin fold being conformed by 8 α-helices (A-H) forming the classical 3/3 sandwich fold ([7] and therein reported citations). In contrast with hemoglobin (HB) and MB, NGB heme iron is hexacoordinated in both its ferrous and ferric forms [7,26,27] with the proximal (His96, F8) and the distal histidine (His64, E7). Therefore, the cleavage of distal His-Fe bound is required for any exogenous ligands bindings [7,26,27,28,29,30,31,32]. NGB reversibly binds oxygen with a P_50_ value ranging from 0.9 mmHg to 10 mmHg for human NGB and from 0.9 mmHg to 2.2 mmHg for the murine globin ([7] and therein reported citations). Since the oxygen binding to NGB is limited by the cleavage of the distal histidine in the hexacoordinated status, the actual oxygen affinity for NGB is considered to be around 1 mmHg such as for MB [7,9,29]. Consequently, data evidencing the low intracellular concentration (~1 μM) of the globin in the total brain ruled out the possibility of a NGB function in the delivery/storage of oxygen in neurons [33]. Despite this, in a pioneering study, Schmidt and collaborators (2003) first evidenced that the NGB levels in retinal extracts from mice were 100-fold higher with respect to total brain extracts estimating a NGB concentration in the retina in the range of 100 to 200 μM, comparable to MB levels in muscle and accountable for a possible NGB function in the transport or short-term storage of oxygen [34]. In the same research, the analysis of the cellular and subcellular distribution of NGB in the retina layers found a strong localization of *NGB* mRNA in the inner segments of the photoreceptors layer, the outer and inner nuclear layers, and in the ganglion cell layer of the retina, whereas lower signals were detected in the plexiform layers and no signal was evidencing in the outer segment of photoreceptor and pigment epithelium [34]. On the other side, the evaluation of NGB protein expression in the retina layers has indicated a differential distribution with respect to those observed for mRNA. In particular, the highest protein levels have been found in both the outer and inner plexiform layers, probably due to a redistribution of the NGB protein after translation, whereas the mRNA and protein expression coincided at the levels of the inner segments of photoreceptors and ganglion cells [34]. Accordingly, other reports have lately shown a comparable NGB distribution in rodent retinal layers [16,35,36,37,38,39] adding a functional connection between NGB and mitochondria as evidenced by the NGB co-localization with mitochondrial proteins [16,37] or the presence of the globin directly inside the organelle [35,38]. In addition, the globin staining at the margin of inner nuclear layers toward the plexiform layer has been hypothesized to have origin by NGB signal from the mitochondria of the synaptic terminals of photoreceptors and horizontal cells [35].

**Figure 1:**
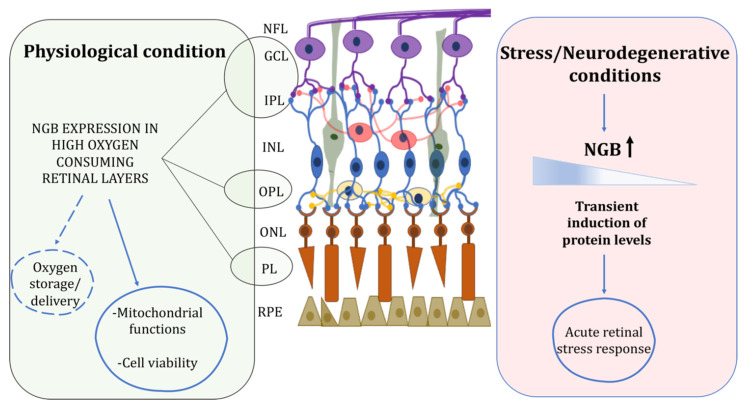
Schematic representation of retinal NGB distribution and proposed functions under physiological and stress/neurodegenerative conditions. In physiological condition, NGB is mainly expressed in high oxygen consuming retinal layers where it could function for oxygen storage/delivery and preservation of mitochondrial functionality and cell viability. Under stress injury, NGB protein levels are transiently induced in retina layers suggesting a role of the globin in the acute stress response. For details, see the text. Retinal pigment epithelium (RPE), photoreceptor layer (PL), outer nuclear layer (ONL), outer plexiform layer (OPL), inner nuclear layer (INL), inner plexiform layer (IPL), ganglion cell layer (GCL), nerve fiber layer (NFL). Dotted lines represent not yet clarified functions.

Overall, NGB distribution in mouse [16,34,36,37] canine [40], rat [16,38,39], and human [41,42] retina has been reported especially in high oxygen-consuming layers of the tissue including photoreceptors, plexiform and ganglion cell layers, whereas in zebrafish retina, under normal conditions, *NGB* mRNA has been reported mainly in the amacrine cells at the INL [43] (Table 1). Additionally, the presence of both *NGB* mRNA and protein were also found in mouse optic nerve and, in particular, in RGCs axons, but also in astrocyte supporting cells [44], shifting the paradigm about the selective expression of NGB in neuronal cell type in brain and retina [41,45,46]. The oxygen binding properties of NGB and its expression pattern closely related to mitochondria and to cells with the higher metabolic rate sustain a major NGB role in oxygen delivery or storage in the retina under physiological conditions where it contributes to the oxygen flow toward mitochondria preventing hypoxia in retinal disease (Figure 1) [16,34,39]. Furthermore, evidence of the expression of NGB just in the outer layers of the avascular retina of guinea pig, the only part where the aerobic metabolism can be sustained and mitochondria are present, sustained the link between NGB and oxygen consumption [16].

However, despite a large pattern of similarities, the analysis of NGB expression and distribution among the retinal layers have also evidenced incongruences among species and experimental conditions (Table 1), showing, sometimes, a very restricted expression of NGB under physiological condition in just some layers and cells type of the retina [42,47]. In addition, Hundal and collaborators in 2012 have reported that in the mouse retina just few neurons of the ganglion layer and inner nuclear layer expressed NGB, strongly questioned any postulated roles of NGB in the retina in terms of both oxygen homeostasis and neuroprotection under normal conditions [25]. Recently, by the construction of a mathematical model for the retinal oxygen distribution, it appeared that the globin is unlikely to play a significant role in oxygen storage, whereas it may be more efficient in the transport during stress hypoxic events than under normal physiological conditions by increasing, for example, the oxygen transport of about the 30–40% in the inner segments of photoreceptor and inner plexiform layer [13].

So that, despite the large number of results obtained over the years, the definition of the exact distribution of NGB and its function in the oxygen metabolism in the retina is yet far to be completely solved. In general, the reported discrepancies on NGB expression in retina layers have relied on the experimental conditions and, in particular, on anti-NGB antibodies specificity.

However, beyond the possible interspecific differences of NGB in the retina, the peculiar property of the globin as an inducible protein whose levels and intracellular distribution is highly modulated by physiological states [48,49], hormones (e.g., 17β-estradiol-E2 [50,51]) and stressing condition [50,52,53,54,55,56,57,58] suggest a strong “plasticity” of NGB protein and its functional outcomes. In addition, because of the vulnerability of the retina and the cellular stress conditions, the definition of a “physiological status” of the tissue could be limited, instead being more variable across individuals and different situations, possibly affecting the NGB levels and distribution. In this context, it has been reported that retinal NGB is modulated in terms of expression, protein levels, and distribution under stressing and pathological conditions. NGB is rapidly overexpressed under hypoxia/ischemia insults in rats [59] and mice [37], after optic nerve injury in mice [47] and zebrafish [42], elevated intraocular pressure (IOP) [36] and blue (453 nm) light exposure [60] in rodent models, although the expression of the globin decrease to the normal levels [36,43,59,60] or at lower levels at longer time of the insult [37,47,61], at least in part due to the stress-induced retinal cell death (Table 2). Indeed, the expression of NGB has been demonstrated to significantly drop in parallel with retinal cell death after chemical-induced degeneration of photoreceptors [62,63] or in the mouse *Harlequin (Hq)* strain which shows defects of mitochondrial respiratory chain complex I and RGCs loss [35]. On the other side, just few human studies about the connection between NGB levels and retinal disease have been yet performed. Among them, the increase in NGB levels in both retina [42] and plasma [64] of patients with advanced chronic glaucoma has been reported proposing the high globin expression as a biomarker of glaucoma disease. On the other side, a plasma NGB downregulation in patients with diabetic retinopathy [65] has been reported suggesting the use of NGB as an inverse biomarker with respect to what is observed in glaucoma.

Consequently, since the inducible nature of NGB and the proposed function in the rapid stress response aimed to preserve retinal tissue [59,60], NGB levels and intracellular/extracellular distribution could differentially reflect the retinal status and the stressing condition (Figure 1). Therefore, the exact correlation between NGB and retinal status is worth to be further deepened for defining NGB functionality in the retina and its possible role as a general biomarker of retinal disease.

## 4. Neuroprotective Effect of NGB in Retinal Disease

The preferential expression of NGB in the retina suggests that it can be sensitive to multiple retinal diseases. Based on the time-dependent modulation of retinal NGB levels under stressing conditions (Table 2), the globin might play a key compensatory neuroprotective function in preserving retinal cells under acute stress injury, acting in the rapid cell stress response that, in turn, could be potentially linked to the use of NGB as a disease marker at the early stages (Figure 1) [59,60]. From this perspective, the research went beyond the definition of the globin expression under normal and/or stressing conditions, focusing on the effect of NGB levels manipulation to explore the physiological and potential neuroprotective function of the globin in retinal tissue damage and diseases. Lechauve and co-workers reported the connection between the NGB knock-down and the decrease in RGCs survival in vitro, and the reduction in nerve fiber density, the overall number of RGCs, and functionality of the mitochondrial respiratory complex I and III accompanied with a visual function impairment in vivo [44]. Based on the obtained data, authors claimed a critical role of endogenous NGB in the maintenance of mitochondrial function and, in turn, of RGCs integrity and viability under physiological conditions [44].

Despite studies reporting no effects of lack of NGB on light-induced cellular stress response [66] have raised doubts over the functional role of endogenous NGB in retinal physiology, several reports demonstrated a function of NGB exogenous overexpression in preventing retinal disease and/or in maintaining retinal structural and functional integrity upon stress exposure. Intriguingly, NGB overexpressing mice exposed to retinal ischemia-reperfusion (IR) injury showed significant preservation of retinal thickness [37]. At subcellular levels, overexpressed NGB localized at mitochondrial levels in ganglion cells, INL and ONL where it exerted a neuroprotective and anti-apoptotic effect under IR conditions by inhibiting the mitochondrial oxidative stress and caspase-3 activation [3]. This evidence sustains the idea that, as well as in brain neurons (for review see [7,8]), up-regulated NGB could directly affect the apoptotic mitochondrial pathway. Furthermore, in glaucoma mice models characterized by the genetic (DBA/2J mice) or experimental induction of intraocular hypertension, the tight connection between NGB mitochondrial location and neuroprotective effect has been demonstrated [36,67]. Indeed, the ability of overexpressed NGB to increase the activity of mitochondrial complex I and III, accompanied with attenuation of ROS production and increase in mitochondrial respiration recovery have been related to the protective function of the globin in preventing RGC loss and nerve fibers disappearance under glaucomatous neuronal damage [36,67]. In the mice model of glaucoma induced by experimental ocular hypertension, NGB-induced RGCs survival was not correlated with the preservation of visual pathway [36]. However, in the genetic model of glaucomatous injury, gene therapy for inducing NGB overexpression improved the cortical response to light stimulation in both mice treated before the beginning of progressive degeneration (2-months old mice) or at an advanced stage of the disease when the neuronal loss has begun (8-months old mice) [67]. In the latter mice, the late treatment for inducing NGB overexpression has been reported to just preserve the remaining RGCs, sustaining a main role of NGB in the protection of retinal neuronal cells promoting their mitochondrial functionality to keep visual function in spite of previous reduction in neuronal cells [67]. Consistently, in *Hq* mice, overexpressed NGB has been demonstrated to restore the mitochondrial function and improve RGCs survival, to maintain nerve fiber integrity, reduce the glial cell activation and, in turn, protect the visual functions [35]. Furthermore, similar effects were also observed in mice treated with N-Methyl-N-nitrosourea (MNU), an alkylant toxicant that causes the selective photoreceptor apoptosis in the mammalian retina, where the induction of NGB overexpression through an adeno-associated-virus vector ameliorated the oxidative stress and apoptotic cell death in photoreceptor cells alleviating the morphological alteration of the retina and the visual impairments [62].

In addition to the transgenic-induced overexpression of NGB, an alternative approach using the injection in the mouse eyes of a chimeric human-zebrafish NGB with the ability to cross the plasma membrane, demonstrated that high levels of intracellular NGB led to a threefold increase in survival of RGCs accompanied with a significant axon-regenerating outgrowth after optic nerve injury [47]. Similarly, the intravitreal injections of mammalian exogenous NGB have been described to restore the long-time decrease in endogenous NGB after 7 days of hypoxia injury and to functionally prevent the stress-induced ganglion cell death and microglial activation, proposing the NGB injection as a potential therapy for retinal disease [61]. Of note, the latter results have been correlated with the intra-retinal penetration of exogenous mammalian NGB due to the small size of the globin and they could not exclude the possibility of a direct extracellular effect of NGB in the retinal tissue. Indeed, despite the NGB ability to cross the membrane and to be released outside cells have been reported just for the zebrafish globin, it has been recently shown the presence of NGB in astrocyte-derived exosomes [68] and the function of sub-nanomolar exogenous NGB concentration in protecting astroglial cells from oxidative stress and cell death [69]. Such new evidence strengthens the idea that mammalian NGB function is not limited to the intracellular context but could be spread outside in the extracellular milieu functioning as an intercellular factor under different stimulation as it occurs in breast cancer [70], widening the vision about the NGB effects under stressing condition also in the retinal tissue.

## 5. NGB Level Modulation as Therapeutic Approach in Retinal Disease

Despite the efficacy of genetic and molecular approaches in up-regulating intracellular NGB levels and enhancing its neuroprotective effects, the identification of exogenous or endogenous inducers of NGB has been considered a valuable way to understand the molecular pathways regulating NGB levels and, in turn, to deepen the NGB neuroprotective functions aimed to preserve neuronal functionality under stressing conditions. In vitro and in vivo evidence have demonstrated the positive effects of stressing condition in regulating NGB levels, justifying a possible role of the globin as a key compensatory factor in the endogenous neuroprotective pathway(s) devoted to promoting adaptive response and resistance to death in the brain neurons [7,58,71,72,73,74], as well as in retinal cells (see above). In addition, several hormones/growth factors (e.g., E2, erythropoietin, EPO; VEGF, thyroid hormone) have been proven to up-regulate NGB in both neuron-derived cells and extra-nervous cancer cells [50,75,76,77,78,79,80,81]. Furthermore, in line with the overarching goal to find out exogenous molecules able to potentiate the NGB neuroprotective function, several natural and synthetic compounds have been reported capable to modulate NGB protein levels [7,82]. Such group comprises iron chelators such as deferoxamine [73] and hemin [63,83], short-chain fatty acids (e.g., Cinnamic and Valproic acid) [80], non-steroidal anti-inflammatory and anti-diabetic drugs [84,85], and a large group of plant derivatives including naringenin, resveratrol, genistein, daidzein, Biochanin A, polydatin, and formononetin [50,86,87]. Although it may be thought that NGB inducers could be effective against retinal neurodegeneration, so far, the study of NGB induction by exogenous compounds as a valuable strategy to potentiate retinal cell ability to cope with stress is quite limited. In this context, Tao and coworkers correlated NGB up-regulation by exogenous inducer with a neuroprotective effect in mice with RP pharmacologically induced by the treatment with MNU. In particular, the authors have indicated that the intravenous treatment with hemin restored the NGB depletion in MNU-treated mouse retina rescuing cone photoreceptor cells and preserving the morphological and functional integrity of the retina, as indicated by the optical coherence tomography (OCT), behavioral and electroretinogram (ERG) examinations. On such results, the up regulation of endogenous NGB by exogenous compounds as a potential tool for the treatment of retinal neurodegeneration has been proposed [63].

**Figure 2:**
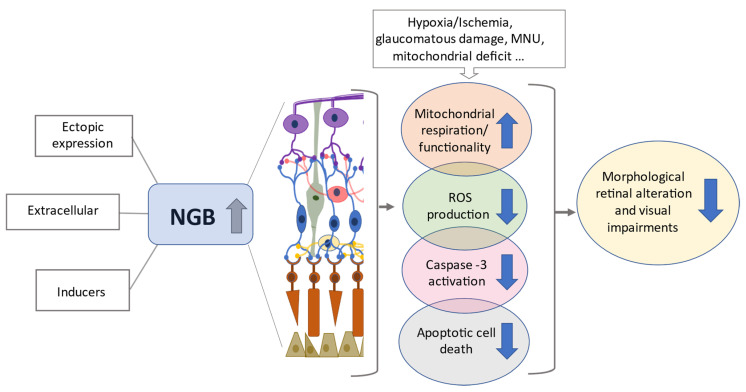
High levels of NGB prevent retinal degeneration. The increase in NGB protein levels in retinal layers due to ectopic overexpression, direct treatment with exogenous NGB or with inducers of intracellular NGB (e.g., hemin), promotes mitochondrial functionality, attenuates oxidative stress and apoptotic cell death (e.g., photoreceptor cells, retinal ganglion cells), preserving, in turn, the visual function under stressing and/or neurodegenerative conditions. For details, see the text. Reactive oxygen species (ROS), *N*-Methyl-N-nitrosourea (MNU).

Evidence of NGB function in preserving the neuronal cell death and visual function when ectopically overexpressed (see above) along with the documented rapid increase in its endogenous levels under acute stress condition [36,37,47,59,60] led to hypothesize that the globin is required as compensatory protein for the rapid cellular response to stress. In this context, it may be thought that any compound able to induce the globin intracellular levels before the stress and/or in a persistent manner, could preserve retinal cells from death and, in turn, degenerative process through a pre-conditioned mechanism (Figure 2).

## 6. Conclusions

The discovery of the intracellular globin NGB has suggested its function as an oxygen supplier and carrier protein in high metabolic active neurons including those of retinal tissue [16,34,39]. Although evidence supports this possibility in the retinal tissue where NGB is 100-fold more expressed [34], over the years, such an idea has been weakened and/or considered to be limited to some retinal layers and specific stressing conditions like hypoxia [13]. On the other hand, a broad literature supports the neuroprotective effects of overexpressed NGB in brain neurons (for review see [7,8]). Despite the differential regulation of retinal NGB level depending on both “timing” and “type” of stressing conditions (Table 2) [36,37,47,59,60,61], wide evidence sustain similar roles of NGB accumulation in the retina. In particular, evidence gathered about the effect of ectopic overexpression of NGB in the retinal tissue, and, in particular, in RGCs, converge to define a critical function of high induced levels of NGB in preserving retinal neuron loss and vision pathway under different stress conditions/retinal diseases as the main consequence of the globin ability to maintain or enhance mitochondrial functions, supporting the idea of NGB as the main target candidate for preserve or restore retinal tissue during degeneration [35,36,37,47,61,67].

Based on the common mechanisms of neurodegeneration across different retinal neurodegenerative diseases including oxidative stress, inflammation, and apoptosis, several therapeutic approaches are aimed at restoring an adequate retinal environment to promote cell viability by using compounds able to impact several biochemical pathways [5]. Among them, the use of known anti-apoptotic agents including inducers of anti-apoptotic proteins or activators of pro-survival pathways (e.g., extracellular signal-regulated kinases ERK) has been successfully proved to preserve retinal structure and function under stressing conditions [5]. Furthermore, the antioxidant and anti-inflammatory functionality of different natural molecules has led to the growing use of such compounds for the treatment and/or prevention of retinal diseases [3,5]. Despite the use of natural compounds for the treatment of different retinal pathologies, experimental evidence indicates that the efficacy of such treatments could not be generalized being dependent on the characteristic and timing of neurodegeneration [3]. For this reason, the proper use of natural compounds and/or the identification of novel similar therapeutic agents might rely on the screening of them for the prediction of their action mechanisms and potential efficacy [3]. In this context, from the data here reviewed two consequences derived: (a) strategies that can up-regulate the expression of NGB are expected to be neuroprotective and, vice versa, (b) the potentiation of NGB accumulation could be a common outcome of neuroprotective agents. In the next future, the identification of novel therapeutic strategies for retinal degeneration and/or the prediction of efficacy of known ones could be addressed by evaluating their impact on NGB levels and functions, opening new perspectives in preventive and/or curative opportunities for retinal disease.

## Figures and Tables

**Table 1:** Summary of NGB mRNA/protein expression in retinal layers among different species.

Retinal Layers	Species	mRNA/Protein	References
RPE	Dog	Protein	[40]
	Human	Protein	[41]
PL Outer segments	-	-	-
PL inner segments	Mouse	mRNA/protein	[16,34,36,37]
	Rat	Protein	[16,39]
	Guinea Pig	Protein	[16]
	Dog	Protein	[40]
	Human	Protein	[41,42]
ONL	Mouse	mRNA	[34]
	Dog	Protein	[40]
	Human	Protein	[41]
OPL	Mouse	Protein	[16,34,36,37]
	Rat	Protein	[16]
	Dog	Protein	[40]
	Human	Protein	[41,42]
INL	Mouse	mRNA	[34]
	Rat	Protein	[39]
	Dog	Protein	[40]
	Human	Protein	[41]
	Zebrafish	mRNA	[43]
IPL	Mouse	Protein	[16,34,36,37]
	Rat	Protein	[16]
	Dog	Protein	[40]
	Human	Protein	[41,42]
	Zebrafish	Protein	[43]
GCL	Mouse	mRNA/Protein	[16,34,36,37,47]
	Rat	Protein	[16,39]
	Dog	Protein	[40]
	Human	Protein	[41,42]
NFL	Rat	Protein	[38]
	Human	Protein	[41]
Optic nerves	Rat	mRNA/Protein	[38,44]

**Table 2:** Summary of NGB mRNA and protein levels modulation by retinal injuries/disease among different species.

Retinal Injury/Disease	Species	Retinal NGB Protein Levels (with Respect to Non-Injured Control)	Retinal *NGB* mRNA Levels (with Respect to Non-Injured Control)	Plasma NGB Levels	References
Hypoxia/Ischemia	Rat	**Increase** 1–15 min after the insults **No change** >20 min after the insults	-	-	[59]
	Rat	**Decrease** at days 7 and 30 post hypoxia	-	-	[61]
	Mouse	**Increase** 12 h after ischemia **Decrease** from da 1 to day 7 after ischemia	**Decrease** from day 1 to day 7 after ischemia	-	[37]
Glaucomatous damage (Elevated intraocular pressure- IOP)	Mouse	**Increase** 3 and 7 days after IOP elevation **No change** thereafter	**Increase** 3 and 7 days after IOP elevation **No change** thereafter	-	[36]
Light exposure	Rat	**Increase** 1–2h after the insult (453 nM blue light) **No change** ≥ 3 h after the insult	**Increase** 2 h after the insults	-	[60]
Optic nerve injury	Mouse	**Increase** 1 day after the injury **Decrease** >2 days after the injury	-	-	[47]
	Zebrafish	**Increase** 3 days after the injury **No change** 7 days after the injury	**Increase** 3 days after the injury **No change** 7 days after the injury	-	[43]
N-Methyl-N-nitrosourea (MNU) (Selective photoreceptor degeneration)	Mouse	**Decrease** from 1 day after the treatment	**Decrease** from 1 day after the treatment	-	[63]
Mitochondrial Respiratory Chain complex I defect and RGCs loss	*Harlequein* mouse strain	**Decrease** in 6-months old mice	**Decrease** in 6-months old mice	-	[35]
Chronic glaucoma	Human	**Increase**	-	**Increase**	[42,64]
Diabetic retinopathy	Human	-	-	**Decrease**	[65]

## Data Availability

Not applicable.
